# Pressure during decision making of continuous sedation in end-of-life situations in Dutch general practice

**DOI:** 10.1186/1471-2296-13-68

**Published:** 2012-07-03

**Authors:** Marco H Blanker, Marlies Koerhuis-Roessink, Siebe J Swart, Wouter WA Zuurmond, Agnes van der Heide, Roberto SGM Perez, Judith AC Rietjens

**Affiliations:** 1 University of Groningen, University Medical Center Groningen, Department of General Practice, P.O.Box 30.001, 9700, Groningen, the Netherlands; 2 Department of Public Health, Erasmus Medical Center, Rotterdam, the Netherlands; 3 EMGO Institute for Health and Care Research, and Departments of Anesthesiology and Palliative Care Centre of Expertise, VU University Medical Center, Amsterdam, the Netherlands; 4 Hospice Kuria, Amsterdam, the Netherlands

**Keywords:** Conscious sedation, Palliative care, General practice, Patient preference

## Abstract

**Background:**

Little is known about pressure from patients or relatives on physician’s decision making of continuous palliative sedation. We aim to describe experienced pressure by general practitioners (GPs) in cases of continuous sedation after the introduction of the Dutch practice guideline, using a questionnaire survey.

**Methods:**

A sample of 918 Dutch GPs were invited to fill out a questionnaire about their last patient under continuous sedation. Cases in which GPs experienced pressure from the patient, relatives or other persons were compared to those without pressure.

**Results:**

399 of 918 invite GPs (43%) returned the questionnaire and 250 provided detailed information about their most recent case of continuous sedation. Forty-one GPs (16%) indicated to have experienced pressure from the patient, relatives or colleagues. In GPs younger than 50, guideline knowledge was not related to experienced pressure, whereas in older GPs, 15% with and 36% without guideline knowledge reported pressure. GPs experienced pressure more often when patients had psychological symptoms (compared to physical symptoms only) and when patients had a longer estimated life expectancy. A euthanasia request of the patient coincided with a higher prevalence of pressure for GPs without, but not for GPs with previous experience with euthanasia. GPs who experienced pressure had consulted a palliative consultation team more often than GPs who did not experience pressure.

**Conclusion:**

One in six GPs felt pressure from patients or relatives to start sedation. This pressure was related to guideline knowledge, especially in older GPs, longer life expectancy and the presence of a euthanasia request, especially for GPs without previous experience of euthanasia.

## Background

Patient pressure is a strong independent predictor of all doctor behaviours [1], and is of importance in decision making in end-of-life situations. Such situations are often complex and involved with emotions, including many decisions to be taken [2]. Beliefs and expectancies from patients as well as relatives, may differ from professional opinions and guidelines [3].

Recently, Swart et al. showed that general practitioners (GPs) involved in the decision making of continuous sedation until death experienced pressure to start sedation from the patient, relatives or others, more frequently than medical specialists and nursing home physicians [4]. In the Dutch national guideline, palliative sedation is defined as ‘the intentional lowering of consciousness of a patient in the last phase of life’ [5]. Continuous sedation until death is the most far-reaching subtype of palliative sedation and is frequently and increasingly applied in home-care palliative patients [6,7]. In the Dutch Guideline, the indication for continuous sedation until death is restricted to an estimated life expectancy of 2 weeks or less. Recently, Hasselaar et al. showed that after the introduction of the guideline, physicians reported that changes in palliative sedation practice conform to the recommendations of this guideline. For example, benzodiazepines were used for sedation more frequently than before and patient involvement in the decision-making process improved [6].

An increasing number of terminally ill patients would prefer to die at home [3], and the majority of GPs consider palliative care to be an important part of their work [8]. Providing palliative care in general practice has some major differences to other settings [8-10]: amongst others, less caregivers are involved in the decision making and the active support of dying patients; and the relation with patients and family members is more intense and long lasting, because of the specific position of GPs as family doctors.

In this study we focus on the decision making to start continuous sedation until death by GPs and the presence of experienced pressure. We wanted to identify both GP and patient characteristics, as well as decision making aspects, associated with the presence of experienced pressure, after the introduction of the national guidelines.

## Methods

We used data from GPs participating in a larger study on continuous sedation in different settings, for which the methods have been described in detail elsewhere [4,11]. In short, in 2008, invitations were send by post to 918 Dutch GPs. Non responding physicians received a paper reminder after two months and an e-mail reminder after four months. Non-respondents (20% random sample) were asked for reasons for non-response. Physicians were asked to fill out a structured questionnaire containing four sections: a. clinical experience with continuous sedation; b. the respondent’s last patient under continuous sedation until death; c. knowledge about and use of the national guideline; d. general information including respondents age, gender, years of experience and having performed euthanasia in the past. Respondents without a recent case of sedation could skip the second part of the questionnaire, as it did not apply.

We included all questionnaires concerning cases of sedation from 2006 onwards in order to reflect the practice of continuous sedation after the introduction of the national guideline in December 2005. We categorized GP practice setting into solitary and group practice, practice areas into urban, mixed, and rural, GP age as younger than 50 years or 50 years or older, and patient diagnosis into cancer and non-cancer.

First, the proportion of separate patients’ symptoms scored as 4–5 (severe) on a 5-point Likert scale was calculated. Next, we classified the symptoms and the decisive indication for continuous sedation as *physical* or *psychological* (Table 1). When more than one symptom was reported, we grouped these symptoms into physical, physical & psychological, or psychological.

**Table 1:** Categorizing symptoms and decisive indications into Physical and Psychological

	
*Symptoms present during decision making*	
**physical**	pain, fatigue, dyspnoe, motoric discomfort, delirium, nausea/vomiting
**psychological**	longing for death, loss of dignity, hopelessness, loss of control, loss of interest, burden to environment and depression
*Decisive indication to start continous sedation*	
**physical**	dyspnoe, pain, physical exhaustion, delirium, nausea/vomiting, motoric discomfort, bleeding, cachexia
**psychological**	existential suffering, anxiety, psychological exhaustion, depression

We estimated the prevalence of experienced pressure by grouping all positive answers for the question “Did you experience pressure before making the decision to start continuous sedation?” which included pressure from the patient, relatives, other health care workers, or other persons. No specific definition of pressure was given. Subsequently, we tested with chi square tests if the following variables were associated with experienced pressure: GP characteristics; patient characteristics; and decision making characteristics. Stratified analyses were performed to test differences of the association between experienced pressure and guideline knowledge for the two GP age groups, using the Mantel Haenszel test. Similar stratified analyses were performed with respect to the association between experienced pressure and euthanasia request for GPs with and without previous experience of euthanasia. Data were analyzed using SPSS 16.0 (SPSS Inc, Chicago, Illinois).

## Results

A total of 399 GPs (43%) returned the questionnaire. Reasons for non-response were: too busy with patient care, no experience with continuous sedation, practiced continuous sedation too long ago, too many requests for participation in research, and never participating in research.

Two hundred-fifty cases were included, as 149 respondents did not report on a case from 2006 onwards. Forty-one GPs (16%, 95% confidence interval 12-21%) reported that they had felt pressure to start continuous sedation from the patient (n = 19), relatives (26) or other persons (7) involved in the care of the patient. The pressure had influenced decision making in 17 (41%) cases.

The GPs characteristics and their relation with experienced pressure are presented in Table 2. GPs with guideline knowledge less often reported experienced pressure than GPs without this knowledge (14% and 25% respectively, *P =* 0.07). Stratified analyses showed that in GPs younger than 50 years, guideline knowledge was not related to experienced pressure (*P =* 0.91), whereas in older GPs there was a strong association: 15% of GPs with and 36% of GPs without guideline knowledge reported pressure (*P =* 0.02); Mantel-Haenszel *P =* 0.094.

**Table 2:** Characteristics of the GPs with included case and relation to experienced pressure

**GP characteristics**	N*	**% within category**	**presence of experienced pressure**	**p-value**^**#**^
Gender	249			0.45
- male		71%	18%	
- female		29%	14%	
GP age (years)	245			0.14
- younger than 50		46%	13%	
- 51 or older		54%	20%	
Area	244			0.15
- urban		48%	19%	
- mixed		12%	25%	
- rural		40%	11%	
Practice	238			0.59
- solitary		24%	19%	
- group		76%	16%	
Ever performed euthanasia	243			0.15
- yes		76%	14%	
- no		24%	22%	
Knowledge about guideline content	240			0.07
- yes		82%	14%	
- no		18%	25%	

* Numbers may differ due to missing values; # p-values reflect chi-square test.

Patient characteristics and their relation with experienced pressure are presented in Table 3. The GPs considered the decisive symptom to be refractory in nearly all cases. In 93% of all cases, the GPs considered the situation to be unbearable at the moment of deciding to use sedation.

**Table 3:** Characteristics of the 250 included cases of continuous sedation until death and relation to experienced pressure

**Patient characteristics**	N*	**% within category**	**presence of experienced pressure**	**p-value**^#^
Gender	245			0.35
- male		54%	14%	
- female		46%	19%	
Age	243			0.06
- 60 years or younger		28%	23%	
- 61 or older		72%	13%	
Main diagnosis	243			0.65
- cancer		85%	16%	
- other, or unclear answer		15%	19%	
Patient was competent	249			0.68
- yes		76%	17%	
- no		24%	15%	
Presence of symptoms during decision making	249			<0.01
- physical only		17%	2%	
- both physical and psychological, or psychological only		83%	19%	
Main indication for continuous sedation	247			<0.01
- physical only		64%	12%	
- both physical and psychological, or psychological only		36%	26%	
Estimated life-expectancy before starting continuous sedation	245			<0.01^$^
- <1 week		72%	13%	
−1-2 weeks		25%	26%	
- >2 weeks		3%	38%	

* numbers may differ due to missing values; # p-values reflect chi-square test; ^$^ Chi-square test linear by linear association.

A strong association was shown between the patient’s symptoms and GP’s experienced pressure: when psychological symptoms were involved, GPs experienced pressure more often, compared to physical symptoms only. Furthermore, a strong association was seen between the patient’s estimated life expectancy at the start of the sedation and experienced pressure.

In 74% of all cases, patients were involved in the decision making before the start of sedation, whereas in 20% patients were only informed about the decision. In less than 6%, patients were not informed at all. Relatives were always informed about the start of continuous sedation, and in 80% of all cases involved.

In 186 cases (77%, Table 4), euthanasia was discussed prior to the decision to use continuous sedation with the patient. If the patient had made a euthanasia request, GPs more often experienced pressure than in cases without such a request (31% versus 13%, *P* < 0.01).

**Table 4:** Decision making characteristics of the 250 included cases of continuous sedation until death and relation to experienced pressure

**Decision making characteristics**	N*	**% within category**	**presence of experienced pressure**	**p-value**^#^
Consultation of palliative care team during decision making	257			0.07
- yes		29%	23%	
- no		71%	13%	
Initiating conversation about continuous sedation	226			0.05
GP		63%	14%	
patient, relative, other		37%	25%	
Euthanasia request expressed by patient	250			<0.01
- yes		80%	31%	
- no (including not discussed)		20%	13%	

Stratified analyses showed that when a GP had previously performed euthanasia, there was no significant difference in pressure between patients with and without a euthanasia request (24% and 12%, *P =* 0.06). If a GP had no previous experience with euthanasia, a euthanasia request coincided with a higher prevalence of pressure (50% vs. 15%, *P =* 0.009; Mantel Haenszel *P* < 0.01).

## Discussion

One in six GPs experienced pressure during the decision making process, by patients, relatives or other persons before starting continuous sedation until death. This pressure had influenced GP decision making in 41%, and was inversely associated with guideline knowledge of older GPs, but to a larger extent associated with patient- and decision making characteristics. More specifically, it was associated with psychological symptoms, estimated life expectancy, GPs not initiating the conversation about continuous sedation, and patient euthanasia requests. For the latter it emerged that GPs who had experience with the performance of euthanasia did not experience more pressure in cases with a euthanasia request.

Experienced pressure in the palliative care situation may result from difficult situations in which patients and their relatives cannot overlook the difficulties that they may encounter, and the wish for a “good death” [9]. Patient beliefs and expectancies, as well as those from relatives, may differ from professional opinions and guidelines [3]. On the contrary, perceived patient pressure may be perpetuated by the physician’s belief in its existence and wish to maintain a good doctor-patient relationship [12]. The latter may be of importance for the palliative homecare setting as well [8]. In contrast to hospital care, GPs more often will prolong their relationship with family members after the death of a sedated patient. For now we can only hypothesize about this, as we did not collect detailed information concerning the reasons why GPs perceived pressure by the patient or relatives. This might be regarded as a possible limitation of our study, and needs further research.

A possible weakness was the low response rate of this study, which is comparable to recent studies in the Netherlands [6,13,14], but lower than earlier studies [7,15], and reflects the difficulties in studying this subject. It is unknown if experienced pressure has influenced the response rate. Therefore, we cannot say if the prevalence of experienced pressure reflects the true prevalence. Also, it is unknown how often physicians refused to provide continuous sedation following a patients’ or relatives request, as only those cases in which continuous sedation was actually applied were included. In one retrospective analyses of medical records in a palliative care unit, the prevalence of “on request” sedation was estimated between 19 and 34% [16]. It was not described whether the physicians perceived pressure in this study.

The strength of our study is that we were able to compare continuous sedation for different practice settings, GP-characteristics, as well as patient characteristics. The latter were similar to those previously described by others, showing mainly cancer patients with an estimated life-expectancy of less than two weeks [6,7,14,15,17]. The majority of patients were sedated because of physical signs or symptoms.

GPs without guideline knowledge more frequently reported pressure. This association with guideline knowledge was strong in GPs older than 50 years, but absent in younger GPs. It is unclear why this age difference is present. Possibly, the attention given to palliative care during the vocational training of Dutch GPs makes younger doctors more aware of possible situations, regardless of specific guideline knowledge. It may be that guideline knowledge makes GPs more comfortable in their reaction to patient questions. Lack of knowledge was identified as one of the barriers to provide palliative care [9]. As in general, continuous sedation is available, patients or relatives may believe that is suitable for individual cases. Starting continuous sedation, however, is essentially a medical decision. Saying no to patients may be difficult in some cases [18]. Guideline knowledge may increase physicians’ confidence when discussing difficult end-of-life issues with patients or relatives.

GPs confidence to respond to situations with patients with merely physical symptoms may also be higher. It appeared that when psychological symptoms were involved, pressure was reported more frequently. It may be possible that psychological suffering elicits pressure. Higher estimated life expectancy coincided with a higher chance of experienced pressure. In the practice guideline, the indication for continuous sedation until death is restricted to an estimated life expectancy of 2 weeks or less [5]. We hypothesize that when GPs estimate a longer life expectancy, they judge that continuous sedation would be too early. When, in such cases, patients or relatives indicate symptoms as unbearable, this may lead to a discrepancy in expectations and may result in discussion, being perceived as pressure by the GP.

When the GP did not initiate the conversation about sedation, they experienced pressure more often. It may be that in such cases, the GP was unexpectedly confronted with a patient request for continuous sedation.

Experienced pressure coincided with a higher number of consultations of the palliative care team before starting continuous sedation. We assume that this higher consultation rate is a result of the perceived pressure rather than a cause of it. In the majority of the consultations, the GPs felt this was of help, which is in line with a previous report [19].

GPs more often experienced pressure in cases with a euthanasia request. This effect was stronger for GPs without previous experience with euthanasia, compared to GPs who had previously practiced euthanasia. This may reflect GPs confidence to respond to patient requests, as was suggested from an earlier qualitative study on the impact of euthanasia on GPs [2]. It may be that in these cases, experienced GPs can better explain why euthanasia cannot be performed, after which the decision making process develops more at ease.

For daily palliative care practice it seems important that GPs have an anticipating role, discussing the possibility of continuous sedation with patients. Physicians are challenged to become aware of the thoughts and expectations of patients and relatives in this important phase of life. Clear communication about end-of-life decisions seems important in an early stage of the palliative care pathway, as well as during continuous palliative sedation, without creating false expectations [20].

## Conclusions

We conclude that one in six Dutch GPs felt pressure from patients or relatives to start continuous sedation until death. This pressure was related to guideline knowledge, especially in older GPs, longer life expectancy and the presence of a euthanasia request, especially for GPs without previous experience of euthanasia.

## Key points

Patient pressure is of importance in decision making in end-of-life situations, such as starting continuous sedation.

One in six general practitioners GPs experienced pressure during the decision making process, by patients, relatives or other persons before starting continuous sedation until death. Pressure was associated with (lack of) guideline knowledge of older GPs, but to a larger extent with patient- and decision making characteristics.

## Competing interests

The authors declare that they have no competing interests.

## Authors’ contributions

MHB participated in the concept and design of the study, acquisition of data, analysis and interpretation of data, drafting of the manuscript, and critical revision of the manuscript for important intellectual content. MK-R participated in the analysis and interpretation of data, drafting of the manuscript, and critical revision of the manuscript for important intellectual content. SJB participated in the concept and design of the study, acquisition of data, and critical revision of the manuscript for important intellectual content. WWAZ and AH participated in the concept and design of the study, obtained funding, and critically revised the manuscript for important intellectual content. RSGMP and JACR participated in the concept and design of the study, obtained funding, acquisition of data, analysis and interpretation of data, drafting of the manuscript, and critical revision of the manuscript for important intellectual content. All authors formed part of the project group for this research and have given final approval of the version to be published. All authors read and approved the final manuscript.

## Ethics approval

According to Dutch policy, the study did not require review by an ethics committee or written informed consent from the patients’ families, because the data collection was anonymous with respect to the deceased patient.

## Funding

This study was supported by a grant from ZonMw and received additional funding of Sint Laurens Fonds Rotterdam and Stichting Palliatieve Zorg Dirksland Calando.

## Pre-publication history

The pre-publication history for this paper can be accessed here:

http://www.biomedcentral.com/1471-2296/13/68/prepub
